# Creating a healthy eating and active environment survey (CHEERS) for childcare: an inter-rater, intra-rater reliability and validity study

**DOI:** 10.1186/s12889-019-7719-8

**Published:** 2019-10-28

**Authors:** Lynne M. Z. Lafave

**Affiliations:** Department of Health and Physical Education, Mount Royal University, 4825 Mount Royal Gate SW, Calgary, Alberta T3E 6K6 Canada

**Keywords:** Early childhood education, ECEC, ICC, Nutrition, Healthy eating, Physical activity, Audit tool, Validation, Intra-rater reliability, Inter-rater reliability [10 keywords]

## Abstract

**Background:**

The CHEERS is a self-administered tool to measure gaps, weaknesses, and strengths of an early childhood education and care (ECEC) centre-based nutrition and physical activity environment. ECEC settings have the potential to profoundly influence early dietary and physical activity behaviours. Content validation of the CHEERS tool has been previously reported. The purpose of this study was to develop reliability and validity evidence for the CHEERS audit tool and the proposed subscales of food served, healthy eating environment, program planning, and physical activity environment in ECEC centre-based programs.

**Methods:**

This cross-sectional study consisted of 2 phases: Phase 1 included inter-, intra-rater and Cronbach’s α. A subset of this sample was invited to participate in a second survey (Trial 2) for intra-rater assessment within 3 weeks of completing the first survey (Trial 1). Phase 2 included concurrent validity assessment between a nutrition expert and the ECEC director using within a one-week period.

**Results:**

One hundred two directors and 85 educators (total of 187) returned the survey. Of these, there were 75 matched pairs for inter-rater reliability analysis providing a CHEERS ICC score of 0.59 and ICC scores ranging from 0.40 to 0.58 for the subscales. The ICC for intra-rater reliability of the CHEERS score was 0.81 for 40 participants completing the survey a second time and a range of 0.72 to 0.79 for the subscales. The CHEERS tool demonstrated very good internal consistency (α = 0.91) and α scores ranging from 0.73 to 0.79 for the subscales. In phase 2, concurrent validation was ICC = 0.65 (*n* = 30) CHEERS scores with a range of 0.42 to 0.69 for the subscales.

**Conclusions:**

This study provides evidence of inter-, intra-rater reliability, internal consistency, and concurrent validity of an environmental assessment audit tool to assess the nutrition and physical activity environment of ECEC centre-based programs. The results demonstrate that the self-administered CHEERS instrument is stable overtime and between evaluators at the same ECEC centre. The scores obtained with CHEERS self-administered audit tool are reasonably accurate compared to an expert rater (dietitian) assessment. This study adds additional support to establishing the psychometric soundness of the CHEERS tool.

**Supplementary information:**

**Supplementary information** accompanies this paper at 10.1186/s12889-019-7719-8.

## Background

Obesity related health issues, such as diabetes and cardiovascular disease, are increasing at alarming rates. This concern is compounded by the rising worldwide prevalence of overweight or obesity in children, a 47% increase from 1980 to 2013 [1], with the recognition that overweight and obese youth track into adulthood [2, 3]. The Ending Childhood Obesity (ECHO) World Health Organization (WHO) report identifies childhood obesity as a complex, under-recognized public health issue where children become obese as a result of entrapment by contextual factors [1]. The early years (0–5 yrs) of childhood are a sensitive period in child development where the origins of obesity can be traced pointing to it as a critical period in which to influence healthy outcomes [1, 4, 5]. An increasing trend in the use of child care in Canada has been observed over the last three decades with over half of Canadian parents (54%) of young children (0–5 yrs) reporting the use of childcare [6]. Therefore, early childhood education and care (ECEC) centre-based programs are an excellent target for public health education and promotion strategies to reach a significant percentage of this population.

There are a number of variables that may influence ECEC nutrition and physical activity environments. Regulatory policy, program resources, physical space, and ECEC practitioner’s knowledge, values, and beliefs regarding healthy eating and activity all influence the ECEC centre’s environment [7–10]. Regulatory oversight for licensed centres exerts a strong environmental influence and policies at the international, national, and regional levels help shape and enhance child environments. Internationally, the WHO ECHO report calls for governments and stakeholders to improve child environments to reduce the risk of obesity [1]. One of the priority areas is early childhood and among these, the directive to provide educators in formal child-care setting with clear guidance and support to encourage healthy eating, sleep, and physically activity habits. The national food guide provides general information for this population and encourages healthy foods but does not provide distinct messaging to the formal child-care setting [11]. The Canadian Society for Exercise Physiology (CSEP) provides national guidance on activity throughout the lifespan. The CSEP early years movement guidelines provide recommendations on physical activity, sedentary behaviour, and sleep combinations for children 0–4 years to achieve a ‘healthy day’ [12]. Specific strategies to implement and apply these guidelines are not included and require the user to interpret and plan activities. One example of a regional policy implementation is the Alberta Nutrition Guidelines for Children and Youth (ANGCY) document. The ANGCY is a comprehensive set of recommendations for healthy foods, servings specific to this age group, a food-rating system to evaluate food choices, guidance on environments to support healthy food choices, and practical examples [13]. However, the guidelines are non-mandatory and in the context of competing demands for practitioners’ time and attention, the reading and implementing of the guidelines is unknown. Cole and colleagues [14] found that Australian ECEC educators utilize personal nutrition knowledge rather than national guidelines when implementing food and nutrition activities with children. Personal nutrition knowledge of ECEC educators may be fraught with misconceptions or inaccuracies which could negatively impact the nutrition environment [15]. One way to enhance the use of nutrition and physical activity guidelines is to provide an audit tool targeted to ECEC educators that encourages assessment, reflection, and awareness of evidence-based practice guidelines in a simplified and user-friendly format.

The CHEERS tool is a self-administered survey that assesses the nutrition and physical activity environments by early childhood educators in ECEC centre-based programs [16] (see Additional file 1). Evidence of content validity for the CHEERS tool has been published, but the tool has yet to undergo reliability testing. The iterative assessment of the validity, reliability, and responsiveness of a health-related end user-reported outcome measure is vital to the development of a high-quality evaluation tool [17, 18]. Guidelines for scale development require evidence of evaluation for use [17, 19, 20]. A systematic review of audit tools assessing physical activity and healthy eating environments in ECEC settings identified a lack of reliability and validity studies of the published tools available [7]. Audit tools need to be constructed well. This is needed for confidence of use by public health professionals and researchers. In a systematic review assessing physical activity and healthy eating environmental audit tools it was reported that this was not always done or done well [7]. The purpose of the current research study was to examine the psychometric properties of the CHEERS audit tool in the context of centre-based ECEC centres.

## Methods

### Instrumentation – the CHEERS tool

The CHEERS audit tool has been designed to offer ECEC centres an evaluative measure for eating and activity environments in an early childhood education context. Evidence of content validity for the CHEERS tool following a rigorous process with an expert panel has been described previously [16]. In addition, the overall tool was determined to have a readability score of grade 8.1 (Flesch–Kincaid) which contributes an important psychometric component of a self-assessment surveillance instrument as accuracy of responses rely on respondent comprehension. The CHEERS audit tool includes 59 items with four proposed subscales (subscales herein): food served (23 items), healthy eating environment (18 items), healthy eating program planning (6 items), and physical activity environment (12 items). Confirmatory factor analysis has yet to be completed for these four subscales. The CHEERS score is calculated by a cumulative average of the four subscales (score range 4–28). The four subscales scores are calculated using an average of the items in the grouping. Each item is measured with a 7 point scale to optimize response discrimination and facilitate appropriate research conclusions [19, 21–23]. Of the 59 items, 86% of these have the response options: always (score = 7), usually (score = 6), frequently (score = 5), half the time (score = 4), occasionally (score = 3), rarely (score = 2), and never (score = 1). The response options for policy items are specific to availability relative to child enrollment in the ECEC centre (week–year). Frequency scales are employed to measure provision of health promotional materials to parents and professional development opportunities for ECEC practitioners. The option ‘do not know’ (score = 1) is included to provide respondents an option to preclude guessing.

### Research design

This cross-sectional study consisted of two phases. In phase 1, there was a focus on inter-, intra-rater reliability, and internal consistency assessment of the CHEERS audit tool with a sample of ECEC centre directors and educators. In phase 2, the focus was on concurrent validity with a sample of ECEC centre directors and an expert external dietitian evaluator.

### Sample

In phase 1, a convenience sample included directors and educators from ECEC centres throughout Alberta. Licensed centres are identified as Day Care Programs (Schedule 1 of the Childcare Licensing Regulation) and are facility-based centres that serve infants, toddlers and pre-school-aged children. They typically provide care throughout the day, from the morning to early evening. To be eligible for the study, centres had to provide care for a minimum of 15 preschool aged (3–5 years) children with the classification of day care program, as opposed to family day home or after school care program. A sample size of 100–200 participants was identified for phase 1 based on an anticipated response rates ranging from 40 to 80% [6, 24–26]. In phase 2, a convenience sample of ECEC centres, with the same criteria as described above were recruited from one large urban location for director participation and centre-based direct observation. A sample size of 30 centres was identified for phase 2 [27].

### Ethics review

This study was approved by the Mount Royal University Human Research Ethics Board (HREB-2011-53d). The participants, ECEC educators and directors, were fully informed about the purpose and procedures of the study and provided written consent for participation. No minors were involved in this study.

### Phase 1: reliability

ECEC centres were randomly selected for recruitment using postal codes to stratify selection of ECEC centres from large urban population centres (population > 100,000), medium population centres (30,000-99,000), and small population centres (1000-29,999), and rural area (population < 1000) throughout five provincial health zones [28]. Centre directors were contacted by phone, provided with a brief summary of the research, and invited to participate in the study. Those agreeing to participate received a package with instructions, consent form, CHEERS survey, demographics survey, and contact information of a trained research associate to answer potential participant questions.

#### Inter-rater reliability

Inter-rater reliability provides a metric for consistency between rater’s scoring of the same environment to provide an estimate of measurement for any two raters are in using a tool [19]. The centre director and one nominated educator completed the CHEERS tool (two members, one-time point survey completion). Directors and educators were instructed to complete and return surveys independently in individually pre-addressed stamped envelopes.

#### Intra-rater reliability

Intra-rater reliability provides a metric for rater’s self-consistency in the scoring which is important in a self-administered assessment tool [29]. Intra-rater reliability investigation required respondents (a subset of raters) to complete the CHEERS survey on two separate occasions (one-member, two-time point survey completion). The interval of repeated survey measurement was within 3 weeks.

#### Internal consistency

Internal consistency provides an understanding of the interrelatedness of test items [30]. Cronbach α, as a measure of internal consistency, was calculated for the overall CHEERS score and each subscale using data from the educators and director’s first survey submission.

### Phase 2: validity

#### Concurrent validity

Concurrent validity provides a type of criterion validity that tests a new tool against existing measures or gold-standard at the same time [19]. In the absence of both, expert observation by a dietitian was considered a gold standard for comparison against the survey completed by the ECEC director.

ECEC centres were recruited from a large urban population location utilizing postal codes to ensure a broad representation of district demographics across the municipality. Centre directors were contacted by phone, provided with a brief summary of the research, and invited to participate in the study. For those agreeing to participate, basic demographics were obtained during this initial screening call. Upon agreement to participate, directors received a package with instructions, consent form, CHEERS survey and contact information of a trained research associate to answer potential participant questions. Each centre’s director completed and returned the survey within 1 week of the scheduled observation.

#### Centre observation visit

A community dietitian working in the population and public health branch of the local health region conducted a one-day site visit at each centre. The same dietitian completed the CHEERS tool for each of the 30 ECEC centres. The subscale observations took into consideration foods served at meal and snack observed, interaction between caregivers and children, policy documents available on display or upon request of director, activity levels in structured and unstructured periods throughout the observation.

A training session between the principal investigator (LL) and the dietitian was conducted to provide orientation to the CHEERS tool, clarify the purpose of the observation, and verify the data collection methods. The same dietitian made all visits which last approximately six-hours, spanning one meal and one snack time for each ECEC centre. The naturalistic observation was structured to be as minimally invasive as possible, with intention not to interfere with normal practice of the ECEC educators. A scheduled visit with the director was arranged in advance to collect information and copies of policy documents relating to healthy eating and physical activity within the observation time.

### Statistical analysis

All data were analyzed using SPSS statistical package version 23 (SPSS Inc., Chicago, IL). Descriptive statistics were used to report means and variation between trials and raters for the overall CHEERS score and subscales. Inter-rater and intra-rater reliability were assessed using intraclass correlation coefficient (ICC) estimates and their 95% confident. This statistic takes into account the selection of raters as well as the correlation and agreement between raters [27, 31, 32]. ICC scores for inter-rater reliability between the ECEC centre director and early learning educator were based on two-way random effects, absolute agreement, single rater/measurement [ICC [1, 2]]) [27, 31, 32]. The ICC scores for intra-rater reliability were based on two-way mixed effects, absolute agreement, single rater/measurement [ICC [1, 3]] [27, 31, 32]. The Cronbach α reliability coefficient was employed as a measure of internal consistency. Streiner and colleagues categorizations were used to interpret α scores: internal consistency values between 0.70 and 0.80 were considered appropriate for the measure of internal consistency [19]. Concurrent validity was assessed by inter-rater agreement of CHEERS scores between the expert rater (dietitian) and centre director based on two-way random effects, absolute agreement, single rater/measurement [ICC [1, 2]] [27, 31, 32]. There are many variations on guidelines for interpreting ICC values. In this study, interpretations of ICC results followed Koo and Li’s categorizations: poor (less than 0.5), moderate (0.5–0.75), good (0.75–0.9), and excellent (greater than 0.90) [27].

## Results

### Sample

In phase 1, 102 directors and 85 educators from the five health care zones throughout Alberta, Canada returned surveys (69% return rate). In phase 2, a total of 43 ECEC centres from a large urban population were approached to participate in the one-day observation site visit and 30 centres consented to participate (70% participation rate).

### ECEC and participant characteristics in phase 1

Directors (*n* = 102) provided information on centre characteristics. On average 53% of ECEC participant centres were not-for-profit and 47% from for-profit with an average child capacity of 68 per centre (Table 1). Most centres provided food to children in their care in some combination of meals, meals and snacks, or snacks. Geographically, centre participation represented the population distribution with a concentration of most centres from large urban population locations and fewer from rural areas. Directors and educators were predominantly female (98%, data not shown), with 10.6 and 7.1 years of experience, respectively (Table 2). Most directors (64%) held a two-year diploma Child Development Supervisor designation. Directors tended to be older fairly evenly spread between 25 and 64 years of age while educator ages concentrated in younger age groups. The education achieved by ECEs was predominantly (46%) the Child Development Assistant (50-h orientation course work), with the remaining achieving the two-year diploma CDS (21%), or a University degree (27%).

**Table 1:** Characteristics of ECEC centres (N or Mean ± SD)

	Phase 1 (*n =* 102)	Phase 2 (*n =* 30)
Number of children enrolled	68 ± 35	70 ± 36
Auspice		
Non-profit	54	15
For-profit	48	15
Food Provision		
Centre provides food	89	26
Parent provides food	13	4
Service geographical location		
Large urban population centre	49	30
Medium population centre	29	
Small population centre	22	
Rural area	2

**Table 2:** Characteristics of ECEC staff

	Phase 1		Phase 2
	Director (*n =* 102)	Educator (*n =* 85)	Director (*n =* 30)
Years in practice	10.6 ± 8.3	7.1 ± 6.1	13.6 ± 9.9
Highest Education Achieved			
CDA - Orientation Coursework	6	39	0
CDW - 1-year certificate - ECEC	3	5	1
CDS - 2-year diploma - ECEC	65	18	21
University degree	28	23	8
Age (years)			
18 and under	0	4	0
19–24	0	33	0
25–34	25	29	8
35–44	29	11	9
45–54	27	5	6
55–64	21	3	7

Note: Years in practice min/max directors = 1–30; educators 1–30

### ECEC and participant characteristics in phase 2

Directors (n = 30) provided information on centre characteristics. Centre participation in phase 2 was split evenly between not-for-profit and for-profit centres with an average capacity for 70 children enrolled (Table 1). The average experience of directors was 13.6 years with 70% holding a two-year diploma (Table 2). Direct observation was completed by a registered dietitian with 20 years of experience and 10 years of public health employment with the health region.

### Reliability

#### Inter-rater reliability

One hundred two directors and 85 educators (total of 187) returned the CHEERS survey. Of these, there were 75 matched pairs (educator and director from the same ECEC) with complete data for inter-rater reliability analysis (Table 3). The overall mean CHEERS score (of a possible 28) as determined by directors was 22.3 ± 2.8 with a range of 13.4 to 26.8 compared to educators of 22.2 ± 2.7 with a range of 15.7 to 27.3. Mean subscale centre scores (of a possible 7) were lowest in the healthy eating program planning subscale (4.7 ± 1.2; 4.8 ± 1.1) and highest in the healthy eating environment (6.1 ± 0.7; 6.2 ± 0.6). The inter-rater reliability for the matched pairs CHEERS score was ICC of 0.59 (CI = 0.41; 0.72). ICCs ranged from 0.40 to 0.58 for the subscale of foods served, healthy eating environment, program planning and physical activity environment.

**Table 3:** Inter-rater reliability for the CHEERS tool

	Directors (mean ± SD)	Educators (mean ± SD)	ICC [95% CI]
Foods Served	5.9 ± 0.5	5.9 ± 0.6	0.54 [0.35; 0.68]
Healthy Eating Environment	6.1 ± 0.7	6.2 ± 0.6	0.42 [0.22; 0.59]
Healthy Eating Program Planning	4.7 ± 1.2	4.8 ± 1.1	0.58 [0.41; 0.72]
Physical Activity Environment	5.5 ± 0.9	5.4 ± 0.8	0.40 [0.20; 0.57]
CHEERS Score	22.3 ± 2.8	22.2 ± 2.7	0.59 [0.41; 0.72]

#### Intra-rater reliability

The ICC for intra-rater reliability was calculated for 40 participants completing the CHEERS survey (i.e. Trial 1) a second time within 3 weeks (i.e. Trial 2). The results are presented in Table 4. The overall CHEERS score for raters in Trial 1 was 21.4 ± 3.0 with a range of 13.4 to 26.7 and 22.3 ± 3.2 with a range of 13.0 to 27.6 in Trial 2. Mean subscale scores (of a possible 7) were lowest in the healthy eating program planning subscale (4.3 ± 1.2; 4.6 ± 1.3) and highest in the healthy eating environment (5.9 ± 0.7; 6.1 ± 0.8). The intra-rater reliability for the CHEERS score was ICC = 0.81 (CI = 0.60; 0.90). ICCs ranged from 0.72 to 0.79 for the subscales of foods served, healthy eating environment, program planning and physical activity environment.

**Table 4:** Intra-rater reliability for the CHEERS tool

	Trial 1 (mean ± SD)	Trial 2 (mean ± SD)	ICC [95% CI]
Foods Served	5.8 ± 0.6	5.9 ± 0.7	0.73 [0.52; 0.85]
Healthy Eating Environment	5.9 ± 0.7	6.1 ± 0.8	0.79 [0.63; 0.89]
Healthy Eating Program Planning	4.3 ± 1.2	4.6 ± 1.3	0.72 [0.52; 0.84]
Physical Activity Environment	5.3 ± 0.9	5.6 ± 1.0	0.74 [0.54; 0.86]
CHEERS Score	21.4 ± 3.0	22.3 ± 3.2	0.81 [0.60; 0.90]

#### Internal consistency

Cronbach α reliability coefficients were calculated for CHEERS score and each of the subscale (Table 5). Returned surveys with incomplete or missing data were removed. The interrelatedness of items in the CHEERS score was α = 0.91. Scores for the subscales were all greater than 0.70: foods served (α = 0.79), healthy eating environment (α = 0.76), healthy eating program planning (α = 0.77), and physical activity environment (α = 0.73).

**Table 5:** Internal Consistency for the CHEERS tool

	Items (n=)	Cronbach α	Sig.
Foods Served	23 (148)	0.79	.000
Healthy Eating Environment	18 (187)	0.76	.000
Healthy Eating Program Planning	6 (187)	0.77	.000
Physical Activity Environment	12 (187)	0.73	.000
CHEERS Score	59 (148)	0.91	.000

Note: Listwise deletion based on all variables in the procedure

### Validity

#### Concurrent validity

Data from 30 dyads (dietitian and a director for each centre) provided a measure of validity. Results are shown in Table 6. The overall mean CHEERS score (of a possible 28) as determined by directors was 22.4 ± 1.7 compared to direct observation of 21.0 ± 1.7. Mean subscale scores (of a possible 7) were lowest in the healthy eating program planning subscale (4.9 ± 0.8; 4.4 ± 0.9) and highest in foods served (6.1 ± 0.5; 6.0 ± 0.5). ICC for the concurrent validity of the CHEERS score was 0.65 (CI = -0.07; 0.88). The subscales of food served and healthy eating program planning resulted in agreements of ICC = 0.69 and ICC = 0.67, respectively. The subscales of healthy eating environment and physical activity environment resulted in agreements of ICC = 0.42 and ICC = 0.51, respectively.

**Table 6:** Concurrent validity for CHEERS tool

	Director (mean ± SD)	Expert Observation (mean ± SD)	ICC [95% CI]
Foods Served	6.1 ± 0.5	6.0 ± 0.5	0.69 [0.45; 0.84]
Healthy Eating Environment	5.9 ± 0.5	5.4 ± 0.4	0.42 [− 0.10; 0.75]
Healthy Eating Program Planning	4.9 ± 0.8	4.4 ± 0.9	0.67 [0.28; 0.85]
Physical Activity Environment	5.5 ± 0.8	5.2 ± 0.8	0.51 [0.19; 0.73]
CHEERS Score	22.4 ± 1.7	21.0 ± 1.7	0.65 [− 0.07; 0.88]

## Discussion

The current study demonstrated good validity and reliability of the CHEERS audit tool in a Canadian child care context. A fundamental goal of an early childhood education is to provide a healthy and nurturing educational environment. ECEC centres require access to validated tools in order to assess their nutrition and physical activity environment for reflection current health practice within their centre. The CHEERS tool can facilitate and empower ECEC centres to enhance nutrition and activity environments that support child health. The CHEERS tool is the first instrument available for ECEC centres in a Canadian context that has undergone rigorous psychometric assessment.

### Inter-rater reliability

The inter-rater reliability measures were moderate for the overall CHEERS tool and two subscales (foods served and healthy eating program planning). The subscales of healthy eating environment and physical activity environment indicated low inter-rater reliability. The reliability findings reported in this investigation are comparable with other healthy eating and physical activity ECEC setting instruments. Inter-rater reliability for the Nutrition and Physical Activity Self-Assessment in child care tool ranged from 0.20 to 1.00 on all questions [33]. Reports of reliability for staff self-report on the ﻿Environment and Policy Evaluation and Observation as a Self-Report instrument for individual components ranged from ICCs of 0.06 to 0.94 [34]. Variability in inter-rater reliability is composed of within-observer and inter-observer differences and this may have resulted for a variety of reasons. Due to the good intra-rater reliability in this study, it is postulated that the differences most likely arise from inter-observer sources. This variability between director and educator may be a result of perspective, education, training, and/or not enough variability in the data. First, educators are primarily aware of activity and practices within their own childcare room. In contrast, the director has a better overall picture of the activity and practices throughout the centre. For example, educators may respond to items asking about educators joining children in active play or sharing information with children about benefits of activity from their own practice where a director’s response may encompass a perspective for all educators in the centre. A second source of variability in the results could be due to the education levels of directors and educators. Directors in the current study predominantly had a 2-year diploma while the educators predominantly were classified as Child Development Assistants which signifies a 50-h education certificate. The educational opportunities to learn about the importance of nutrition and physical activity in the training and formal education differ considerably. A reduced scope in a 50-h educational opportunity would require educators to rely more heavily on personal information, which has been found to be fraught with misconceptions or inaccuracies [15]. Third, training has been identified as a potential modifier to improve inter-rater reliability [19]. Providing training on a survey tool may enable raters to align their interpretation and understanding of items that would provide lower variability of responses. Rater instructions did not include pre-training. Lastly, even when there is substantial agreement among raters, ICC may be low if there is not sufficient variation between cases [27]. These may have contributed to variability observed in this study.

### Intra-rater reliability

The results of our study indicate that the intra-rater reliability of the CHEERS tool is good. The ICC between the first and second CHEERS administration was 0.81 for the overall test score and greater than 0.70 for each of the four subscales. Intra-rater reliability of a scale is a pre-requisite for good inter-rater reliability [19].

### Internal consistency

The internal consistency score for the CHEERS tool and its subscales are appropriate for health measurement scaling. However, an overall CHEERS score of α = .91 indicates there is potential overlap and repetition of items. Future research will employ a factor analysis to reduce the overlap in items.

### Concurrent validity

Concurrent validity was measured in this study comparing ECEC director CHEERS scores to direct observation CHEERS scores completed by an expert rater. Results indicate that reporting by directors is reasonably consistent to expert observation. Moderate ICCs were demonstrated for the overall CHEERS score and three of four subscales. The concurrent validity findings reported in this investigation are comparable with other healthy eating and physical activity ECEC setting instruments. In the child-care nutrition and physical activity environment measure, percent agreement between direct observation and director completed survey ranged from 39 to 97% with more than half achieving 80% agreement [35]. Similarly in a measure to evaluate healthy eating and physical activity policies and practices in Australian childcare services, percent agreement ranged from 38 to 100% with almost half achieving 80% or greater agreement and Kappa scores ranging from poor agreement to perfect agreement [36]. In the current study, the food served subscale achieved the highest ICC score. This may be due to the fact that survey items in this grouping are more easily observed such as serving vegetables, meat alternatives, or whole grain products that can be verified through observation or with posted menus. However, agreement was weaker within the healthy eating environment subscale. Items in this grouping include observable actions such as sitting with children during meals and sufficient time to eat, but also include items such as avoiding food as a reward or staff giving input into menus. These elements may not occur every day or may have occurred outside the six-hour visit period and thus be missed. An expert rater cannot evaluate items that are not documented in policy or apparent during observation. Alternatively, it may be the case that an expert rater has higher expectations of compliance with nutrition policy resulting in lower scores. In contrast, the director has a better overall picture of the different aspects throughout the ECEC centre and can use this knowledge when responding. These variations may help to contextualize the variation in the scores reported by directors and expert rater. Similarly, items in physical activity environment grouping include a range of objectively identified items such as equipment for physical activity and outdoor physical activity time that can be observed or reviewed from documentation. However, weekly screen time estimates or physical activity education with children are variable in occurrence or ability to observe in a single day observation visit. Policy statements indicating how these circumstances are handled in the centre can be used for comparison, however not all centres have detailed physical activity policies leading to potential differences. These findings are similar to other validation studies where direct observation and document review was used to assess validity of a childcare centre nutrition and physical activity measure [34, 36]. Higher agreement occurred for common observable practices such as food or beverage availability and lower for more intermittent items such as role modeling and physical activity-based assessment.

This leads to the question of who is best to assess the child care environment for nutrition and physical activity. Expert observation is often viewed as the gold standard for assessment. However, the expert visit assessment is fraught with potential error such as the inability to assess intermittent activities and limitations of generalization of usual practice based on a small sample (single visit). In addition, this approach creates a power imbalance and judgement in a fragile and emerging profession. If the goal of the tool is to enhance the healthy eating and activity environment, a collaborative team approach would help integration of assessment. In a self-report approach, directors and educators have a vast time frame and number of sample days from which to draw information. This approach is not without potential error such as: reporting bias to selectively present the best version of self; introspectively, the ability to fully consider the item response; and underreporting due to perceived or real knowledge gaps. It is critical to ensure participants are aware that data is analyzed in aggregate and no judgement made on the centre. Our concurrent validation findings indicate that directors and expert observer achieved similar assessments in most cases which strengthens the use of the CHEERS tool for assessment of healthy eating and activity in the ECEC setting.

### Limitations

There are a number of limitations in this study. Firstly, in this study two raters were used for reliability analysis, however, whenever possible, three raters are preferred [27]. A better assessment of reliability may have been provided with an additional rater within each centre. Second, the context for the reliability analysis was a province-wide convenience sample which may limit the generalizability of the results to other populations. However, considerable effort was made to identify a selection of centres representing programmatic diversity in economic, child capacity, length of operation, and auspice (for-profit vs not-for-profit) to be as representative of the provincial context as possible. Replication in other contexts and areas is recommended to strengthen the validity of the tool. Third, child care centres that do not serve food to children in their care only complete five of 23 questions in the food served subscale; this limits the items that contribute to this subscale for these types of centres. Fourth, some items were left blank within returned surveys. This led to missing data from 39 surveys. As a result, the Cronbach α was calculated on the remaining surveys (i.e. 148/187) for the food served subscale and the overall CHEERS score. It is possible that the missing data could have changed the internal consistency results. Fifth, survey response options ‘do not know’ and ‘never’ were assessed a score of 1 point. This did not permit analysis to differentiate between ‘do not know’ and ‘never’. This differentiation should be considered moving forward in order to provide more detail on knowledge gaps existing in ECEC professional’s knowledge of nutrition and physical activity practices occurring in their centre. Last, training in the current study was limited to basic instructions of how to fill out the document. A more robust introduction to the tool, an orientation on how to interpret items and selection of responses may have improved inter-rater reliability.

### Future research & implications

An exploratory factor analysis is needed clarify the subscales for the CHEERS score. In addition, given the potential differences between directors and educators, future studies would benefit from evaluating CHEERS survey results between two educators in the same room as a measure of inter-rater reliability. Additionally, an online survey for this community would be valuable as well as some form of immediate feedback to the respondents.

## Conclusions

The current study builds on previous work by further providing evidence of reliability and validity indices for the CHEERS tool in practical settings. ﻿This study utilized established taxonomy, terminology, and definitions used for developing and evaluating health instruments [17]. This enhances the uniformity and confidence of the measurement properties used to assess CHEERS. The results presented provide evidence of acceptable reliability and validity indices for CHEERS to offer ECEC centres an assessment measure for the eating and activity environments in an early childhood education context. This will also be a valuable tool for public health providers and researchers that can be used to measure the environmental context in a child care setting.

## Supplementary information

**Additional file 1:** CHEERS survey questions.

## Data Availability

The datasets used and/or analysed during the current study are available from the corresponding author on reasonable request.

## Abbreviations

CHEERS: creating healthy eating and activity environments survey
CSEP: Canadian Society for Exercise Physiology
ECEC: early childhood education and care
ECHO: Commission on Ending Childhood Obesity
ICC: intraclass correlation coefficient
WHO: World Health Organization
