# Supplementary material for: Creating a healthy eating and active environment survey (CHEERS) for childcare: an inter-rater, intra-rater reliability and validity study
Source: BMC Public Health. 2019 Oct 28;19:1384. doi: 10.1186/s12889-019-7719-8 (PMC6816176; doi:10.1186/s12889-019-7719-8)
Supplement: Supplementary file 1 — Additional file 1: CHEERS survey questions. [file 12889_2019_7719_MOESM1_ESM.pdf]

CHEERS survey questions

Does your childcare centre provide food to children (meals and/or snacks)?

☐ Yes (1) ☐ No (2)

FOODS SERVED

1. My childcare centre serves meals that include foods from each of the four food groups of Canada's Food Guide
2. My childcare centre serves snacks that include foods from two or more food groups of Canada's Food Guide.
3. My childcare centre serves vegetables and fruit prepared with little or no added fat, sugar or salt.
4. My childcare centre serves one orange vegetable each day (click here for examples).
5. My childcare centre serves one dark green vegetable each day (click here for examples).
6. Half of the grain products served at my childcare centre are whole grain products.
7. My childcare centre serves children over 2 years of age at least 1 cup of fluid milk or fortified soy beverage each day.
8. My childcare centre offers meat alternatives such as beans, lentils or tofu at least once per week.
9. My childcare centre limits foods that contain trans-fat (click here for examples).
10. My childcare centre offers foods that provide a variety of colours, shapes, flavours, and textures.
11. My childcare centre introduces new food items to the menu.
12. My childcare centre offers new foods with familiar foods.
13. My childcare centre's menu includes foods from a variety of cultures represented in my childcare.
14. My childcare centre limits foods that are not on Canada's Food Guide (Click Here for Examples).
15. Any time my childcare centre serves food an option from Canada's Food Guide is offered.
16. My childcare centre has enough food available to meet the needs of the children in our care.
17. My childcare centre limits juice (100% juice) to one or less serving per day (125ml or ½ cup or 4 oz).
18. My childcare centre limits sugar-sweetened beverages (e.g. punches, drinks, cocktails, sports drinks, pop, etc.).

Start at This Question: If "Does your childcare centre provide food to children (meals and/or snacks)?" = No

19. My childcare centre considers food allergies and choking hazards before foods are served.
20. Families who send food for their child at my childcare centre follow Canada's Food Guide.
21. Families who provide food to share with children at my childcare centre follow Canada's Food Guide.
22. My childcare centre always has water available for children to drink.
23. My childcare centre offers water as the only beverage between meals and snacks.

HEALTHY EATING ENVIRONMENT

24. My childcare centre provides regular meal or snack times every 2 to 3 hours.
25. My childcare centre gives children 20 to 30 minutes to eat their meals.
26. At my childcare centre, educators encourage children to taste new foods or less favourite foods without threats or bribes.
27. At my childcare centre, educators allow children to decide how much and whether to eat, from the food offered.
28. My childcare centre provides children with an assigned area, with few distractions, to sit and eat.

Display This Question: If Does your childcare centre provide food to children (meals and/or snacks)? = Yes

29. i) At my childcare centre educators eat the same food and drink as the children.

Display This Question: If Does your childcare centre provide food to children (meals and/or snacks)? = No

ii) At my childcare centre educators eat food and drink that follows Canada's Food Guide.

30. At my childcare centre, at least one educator sits and eats with the children at meals and snacks.
31. At my childcare centre educators talk pleasantly with children during meals and snacks.
32. At my childcare centre educators, avoid using food as a reward, threat, bribe or punishment.

All rights reserved (c) 2019. Lynne Lafave. CHEERS for child care is the copyright of the Dr. Lynne Lafave and must not be adapted, modified or translated without permission. For more information, visit: www.cheerskids.ca

33. At my childcare centre educators talk with families about their children eating habits.

Display This Question: If Does your childcare centre provide food to children (meals and/or snacks)? = Yes

34. i) My childcare centre provides opportunities for families to give input into menus.

Display This Question: If Does your childcare centre provide food to children (meals and/or snacks)? = No

ii) My childcare centre provides opportunities for families to discuss healthy meal and snack options.

35. My childcare centre staff members have opportunities to learn about food customs and cultural practices of children in our program.
36. My childcare centre staff members have access to child specific healthy eating resources.
37. My childcare centre staff members use healthy eating resources.

Display This Question: If Does your childcare centre provide food to children (meals and/or snacks)? = Yes

38. i) My childcare centre provides opportunities for staff members to give input into menus.

Display This Question: If Does your childcare centre provide food to children (meals and/or snacks)? = No

ii) My childcare centre supports staff members in speaking with families about healthy meal and snack options.

39. My childcare centre follows a written healthy eating policy.
40. My childcare centre informs families of our healthy eating policy.
41. My childcare centre follows a healthy eating policy when using foods in fundraising.

PROGRAM PLANNING

42. My childcare centre uses meal and snack time as an opportunity for educators to talk with children about characteristics of foods (e.g. colour, smell, texture).
43. My childcare centre provides children with opportunities to experience preparing food.
44. My childcare centre integrates nutrition education into all parts of program planning (e.g. story time, songs, prop boxes, kitchen centres, crafts, field trips, active play, etc.).
45. My childcare centre supports healthy eating through visual displays (e.g. posters, books, pictures, etc.).
46. My childcare centre offers nutrition education opportunities to families (e.g. take home materials such as newsletters, activities, workshops, etc.).
47. My childcare staff members attend professional development on nutrition education.

PHYSICAL ACTIVITY ENVIRONMENT

48. My childcare centre provides daily physical activity opportunities to all children (structured and unstructured play).
49. My childcare centre provides children access to a variety of equipment to engage children in physical activity (e.g. balls, hoops, rings, etc.).
50. My childcare centre has indoor space available for physical activity.
51. At my childcare centre, children spend 30 minutes or more each day outside being physically active (except when extreme weather conditions exist).
52. At my childcare centre, educators join children in physically active play.
53. At my childcare centre, staff members share information with children about the benefits of being physically active.
54. At my childcare centre, screen time (including educational uses) for each child on average occurs……
55. My childcare centre provides all children with physical activity opportunities regardless of behaviour.
56. My childcare centre follows a written physical activity policy.
57. My childcare centre informs families of our physical activity policy.
58. My childcare educators attend professional development on physical activity.
59. My childcare centre offers physical activity education opportunities to families (e.g. take home materials such as newsletters, activities, workshops etc.).

All rights reserved (c) 2019. Lynne Lafave. CHEERS for child care is the copyright of the Dr. Lynne Lafave and must not be adapted, modified or translated without permission. For more information, visit: www.cheerskids.ca
